# Assisted closed-loops for brain-computer interfaces

**DOI:** 10.1186/1471-2202-14-S1-P406

**Published:** 2013-07-08

**Authors:** Jacobo Fernandez-Vargas, Hanns Uwe Pfaff, Francisco B Rodríguez, Pablo Varona

**Affiliations:** 1 Grupo de Neurocomputación Biológica, Dpto. de Ingeniería Informática, Escuela Politécnica Superior, Universidad Autónoma de Madrid, 28049, Madrid, Spain

## 

**Figure 1:**
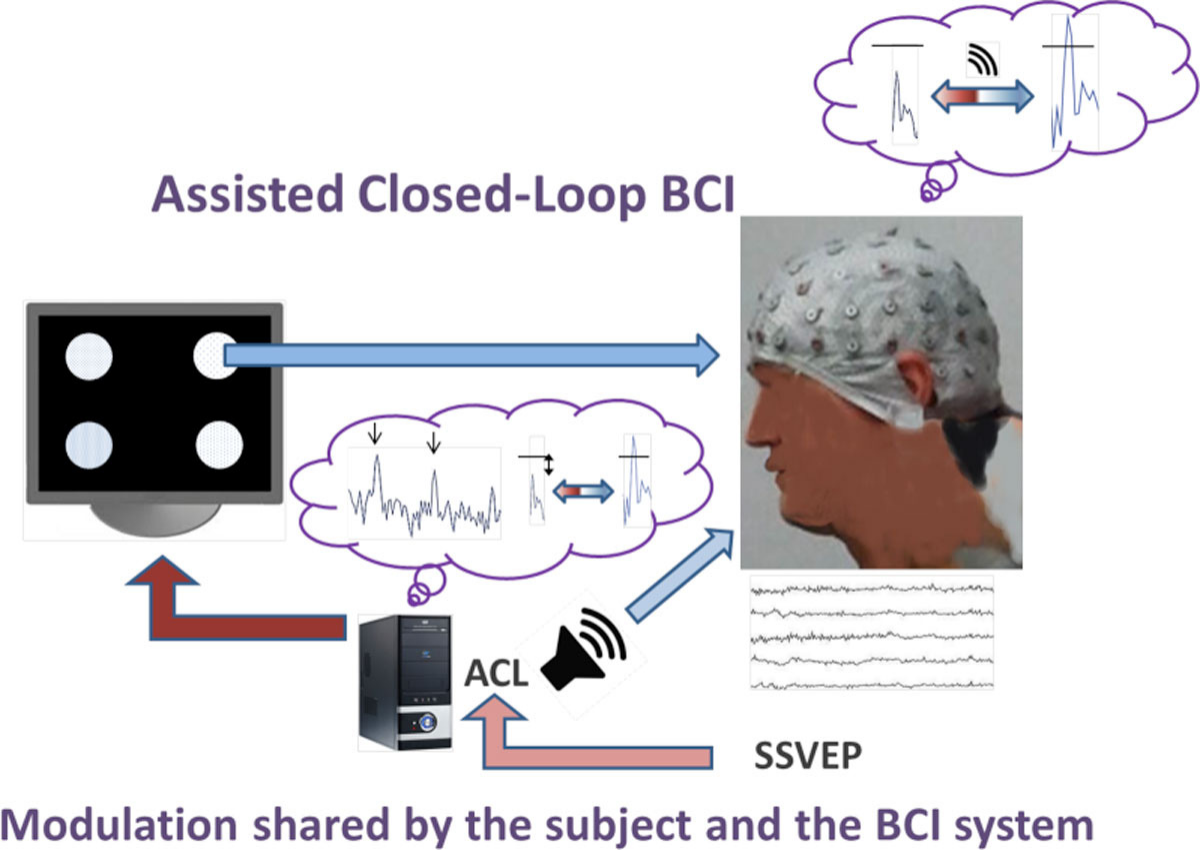
**The assisted closed-loop (ACL) provides online information to the system about the most effective flicker frequencies and to the human subject about the actual distance to the pre-defined SSVEP detection threshold by a continuous auditory feedback (speaker symbol), which leads to an improved control of the BCI**.

The *dynamic clamp *technology to implement artificial membrane or synaptic conductances is one of the most successful examples of closed-loop interactions with the nervous system for observation and control purposes (for a review see [1]). We recently proposed a generalization of the *dynamic clamp *concept to design goal-driven closed-loop interactions with biological systems beyond electrical stimulation and recording in the context of *in vitro *electrophysiology and animal ethology [2]. Following the same activity-dependent stimulation approach, we designed an *assisted closed-loop *(ACL) to optimize the efficiency of brain-computer interfaces (BCI) based on *steady state visually evoked potentials *(SSVEP) [3]. SSVEP based BCIs use multiple visual stimuli such as LEDs or regions on a screen flickering at different frequencies. A subject's brain generates electrical activity at the same fundamental frequency as the visual stimulus on which the subject has focused his/her attention. The ACL consists in the delivery of online information with regard to the control over the given BCI goal both to the human subject and to the system, resulting in an online adaptation of BCI stimuli properties, in this first proof of concept study the combination of the employed flicker frequencies (Figure 1). The optimization process is realized by first performing a real-time closed-loop search for the best set of stimulation frequencies to achieve the given BCI goal, which informs the system about the most effective flicker frequencies to produce SSVEPs in a given subject. Secondly, a continuous auditory feedback informs the subject about the actual distance to a predefined detection threshold.

Our results show that this shared online information leads to an enhanced BCI efficiency, as compared to classic BCI protocols, by helping them to reach the BCI goal in their interaction. The analysis also shows that the ACL protocol takes into account interindividual variabilities. In particular, baseline resting state EEG measures seem to predict subjects' BCI performances, which indicates that the ACL based BCIs might be expanded to innovative new diagnostic/therapeutic tools for clinical contexts and as new paradigms for basic research. Taking all together, these results illustrate that assisted closed-loop protocols can reveal dynamics otherwise hidden under traditional stimulation techniques, provide control of regular and pathological states, induce learning processes, bridge between distinct levels of analysis and lead to automation of experiments. The proposed approach might have a large impact for applied uses, such as computer control and biomedical or prosthetic uses, but also in novel paradigms for neuroscientific and biomedical research.
